# Contractile and Tensile Measurement of Molecular Artificial Muscles for Biohybrid Robotics

**DOI:** 10.34133/cbsystems.0106

**Published:** 2024-05-08

**Authors:** Yingzhe Wang, Kaoru Uesugi, Takahiro Nitta, Yuichi Hiratsuka, Keisuke Morishima

**Affiliations:** ^1^ Department of Mechanical Engineering, Osaka University, Osaka 565-0871, Japan.; ^2^ Department of Mechanical Systems Engineering, Ibaraki University, Ibaraki 316-8511, Japan.; ^3^ Applied Physics Course/Department of Mathematical and Design Engineering, Gifu University, Gifu 501-1193, Japan.; ^4^ School of Materials Science, Japan Advanced Institute of Science and Technology (JAIST), Ishikawa 923-1292, Japan.; ^5^ Global Center for Medical Engineering and Informatics, Osaka University, Osaka 565-0871, Japan.

## Abstract

A printable artificial muscle assembled from biomolecular motors, which we have recently developed, showed great potential in overcoming the design limitations of conventional biohybrid robots as a new bio-actuator. Characterizing its contractility for extending its applicability is important. However, conventional measurement methods are composed of complex operations with poor reproducibility, flexibility, and real-time responsiveness. This study presents a new method for measuring the contractile force generated by artificial muscles. A measurement system was constructed, wherein artificial muscles were patterned by UV laser scanning in an oil-sealed microchamber, and the contractile force was measured in real time using a microforce sensor extended by a 3D-printed microcantilever. The measurement accuracy of the sensor was ensured through calibration and correction. For demonstration purposes, a series of contractile measurements were carried out using the proposed system. The relationship between contractile force and the dimensions of the activation space of the artificial muscles, as well as the tensile properties of the contracted muscle chain were evaluated. The results will help characterize the contractile properties of the artificial muscle and lay the foundations for its further application in biohybrid robotics.

## Introduction

**Fig. 1.**
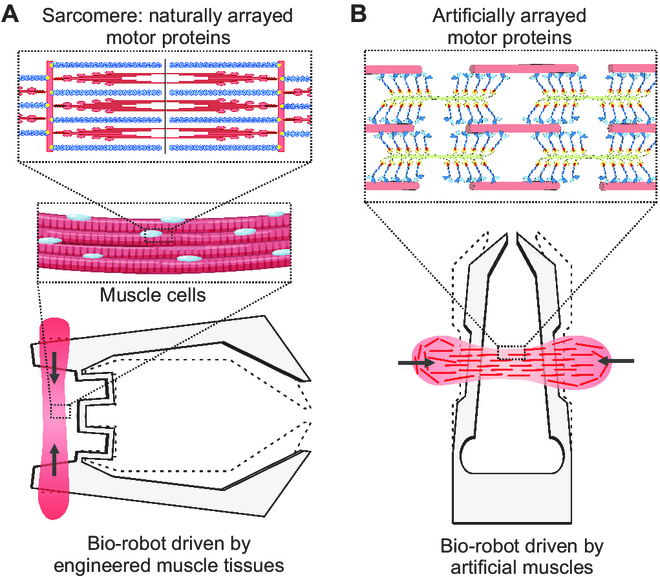
Concept of biohybrid robots, taking two common designs of microgrippers as examples. Bio-robots driven by (A) engineered skeletal muscle tissues composed of living muscle cells and (B) artificial muscles composed of biomolecular motors.

The field of biohybrid robotics has been attracting increasing attention recently owing to its attractive features, such as its eco-friendliness, adaptability, self-healing capabilities, and high energy efficiency [1,2]. Living muscle cells have been widely used to develop various biohybrid robots, such as micropumps [3–5], microgrippers [6–8], and microswimmers [9–11]. These bio-robots are usually constructed through the bottom-up integration of engineered muscle tissues with flexible artificial substrates and transforming the contractions of the muscles into the deformation or movement of the substrates (Fig. 1A) [12]. However, time-consuming cell cultivation and the integration process of muscle cells with artificial substrates have increased the difficulty of manufacturing these bio-robots, thus limiting their applicability. Recently, some progress has been made in directly integrating biomolecular motors into gels and fibers to produce millimeter-scale movement [13,14]. In our previous research, we succeeded in assembling biomolecular motors into a muscle-like contractive network in the desired location through ultraviolet (UV) illumination [15] (Fig. S1). This biomolecular artificial muscle could generate the considerable force needed to drive the actuation of millimeter-scale mechanical components, and a new type of biohybrid robot could be achieved using this novel bio-actuator (Fig. 1B). Compared to living muscles, although this printable artificial muscle lacked the abilities of self-healing from injury, and dynamic response to changing environmental conditions, it was easier to construct and could be integrated more flexibly with mechanical components, highlighting its unique potential in microrobotics [16]. Using optofluidic lithography, soft microrobots driven by such artificial muscle have been fabricated with flexible design and high efficiency [17].

One of the most important functional characteristics of the artificial muscle is its ability to produce contractile forces. Conventionally, the contractile performance of biological muscles is measured using the cantilever-bending method, wherein contractive muscles are mounted on microcantilevers or micropillars, and the contractile force is estimated by measuring the deflection of the cantilevers [18–23]. We have also previously used this method to demonstrate the micronewton-scaled contractile force of our artificial muscle. However, this method largely depends on the mechanical premeasurement and dynamic observation of the cantilever, which requires complex operations and is prone to lags and errors. The measurement reproducibility is also poor because the mechanical properties of cantilevers are usually unstable. Therefore, to achieve a higher stability and temporal resolution, we aim to develop a system for measuring the contractile force of artificial muscles directly using a standard force transducer. In addition, such measurement is more flexible, allowing for the adjustment of contraction conditions, such as the size of the illumination area or the distance between the muscle-anchoring pillars, which helps analyze the influencing factors of artificial muscle contractility.

To achieve such measurement, a force transducer with high sensitivity, strong robustness, and suitable geometry for anchorage to the artificial muscle is necessary. With the development of microelectromechanical systems (MEMS) technology, various microforce sensors, which can be classified into different categories based on the relationships between mechanical forces and sensory properties, including piezoresistive, piezomagnetic, piezoelectric, capacitive, and optical, have been proposed [24]. Among these, capacitive microforce sensing has the advantages of requiring low power, producing low noise, having high sensitivity, and being unaffected by temperature variations. Recently, MEMS-based capacitive force sensors have become commercially available [25] and are been widely applied in mechanically characterizing biomaterials [26–28].

In this study, we used a commercial MEMS capacitive force sensor as the force transducer and extended its sensing part to make it anchorable to the artificial muscles when measuring their contractions. A measurement system was constructed wherein artificial muscles were precisely photo-patterned by UV laser scanning in an oil-sealed microchamber, and the contractile force was directly measured using the extended sensing probe. Calibration tests were performed using a standard microforce sensor with a higher accuracy than that of the sensing probe, and the span error of the probe was evaluated to allow for correction. A series of measurements were conducted using the proposed measurement system to characterize the relationship between the contractile force of artificial muscles and the width and height of the space in which molecular motors were optically activated, since these two dimensions can largely determine the force generation according to our previous research. The tensile strength of the contracted artificial muscle was also tested.

## Materials and Methods

### Measurement system

**Fig. 2.**
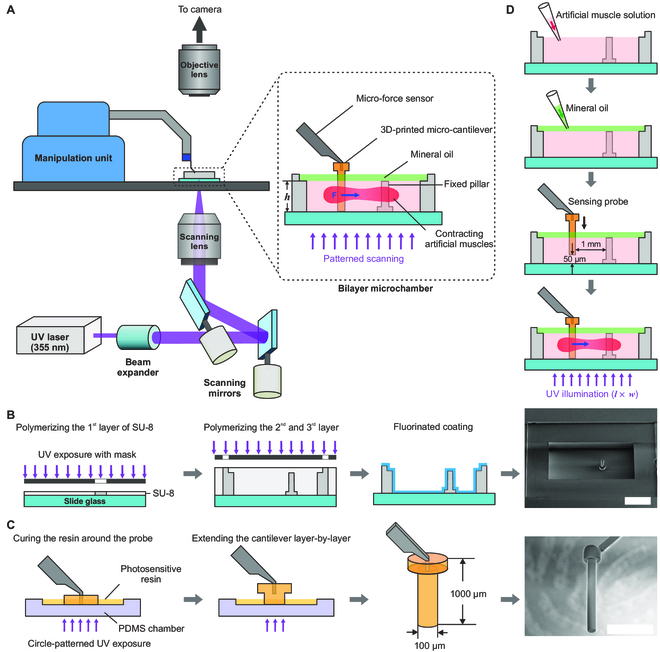
Construction of the contractile force measurement system. (A) Schematic diagram of the system. (Inset) Details of the bilayer microchamber. (B) Fabrication process of the bilayer microchamber and the SEM image of a fabricated one (*h* = 560 μm). One layer of SU-8 was first photo-polymerized by UV exposure as the base of the fixed pillar. Then, the second and third SU-8 layers were polymerized in the same way. After development, the SU-8 microchamber was modified by fluorinated coating. (C) 3D-printing process of the microcantilever to extend the microforce sensing probe and the SEM image of the extended sensing probe. A base of the cantilever was first printed around the sensing probe through circle-patterned UV exposure. Then, the cantilever was extended layer by layer by curing the resin. (D) Process of measuring contractile force. Scale bars, 1 mm (B) and 500 μm (C).

As shown in Fig. 2A, the measurement system comprises a bilayer microchamber on a stage, an extended microforce sensing probe connected to a manipulation unit, a UV scanning system under the stage, and a microscope system above the stage. The manipulation unit (FT-MU, FemtoTools) was used for accurately steering the sensing probe and converting the voltage signal from the sensor to digital signals. The UV scanning system was set up to provide precisely patterned UV illumination. An expanded UV beam from a pulsed laser source (355 nm, UV-F-355-100mW, Ningbo Lasever Inc.) was scanned by a dual-axis galvanometric mirror system (GVS412, Thorlabs) and focused on the middle of the microchamber with an F-Theta scanning lens. The fluorescence microscope system was set up to observe the measurement process. Although not required in general, to avoid any unexpected vibration interference, the entire system was installed on a vibration isolation table. The software system was programmed using LabVIEW for integrated device control and data recording.

The first layer of the microchamber is used as a container for the artificial muscle solution with a length of 4 mm, a width of 2 mm, and a 120-μm-diameter fixed pillar. Its height (denoted as *h*) was controlled from 410 μm to 710 μm as a variable of the contractile measurement. The second layer sealed the microchamber with mineral oil to prevent the solution from evaporating and to decrease the likelihood of unwanted disturbances occurring during measurement. The microchamber was fabricated using a multilayer photolithography process of SU-8 on a glass slide [29] (Fig. 2B). To minimize protein adsorption, the microchamber was hydrophobically modified with a fluorinated coating (Optool HD-1100TH, DAIKIN).

When measuring, contractive artificial muscles were printed in the microchamber with UV laser scanning a rectangle pattern and bridged the fixed pillar and the sensing probe. During contraction, a horizontal tensile force was gradually generated on the lengthened part of the sensing probe and measured as a transduced voltage signal. The signal underwent A/D conversion and was recorded in real time using the software system; the temporal features of artificial muscle contractility could be characterized. Additionally, it was easy to adjust the illumination patterns and geometry of the microchamber for analyzing their effect on the contractility.

### Extension of sensing probe

A commercial microforce sensing probe (FT-S1000-LAT, FemtoTools) with a resolution of 0.05 μN and measuring range from −1,000 to 1,000 μN was employed in this study. It used a differential tri-plate comb drive actuator to achieve high sensitivity and linearity. The vertical force applied to the sensing part of the sensor causes a tiny displacement of the movable capacitor plates connected to the probe, which is proportionally transformed to a voltage signal.

Since the original sensing component of the sensor was only 50 μm long and 10 μm wide, we extended the sensing probe for inserting it into the microchamber and using it as an anchor for the artificial muscles by three-dimensionally (3D) printing a microcantilever structure (Fig. 2C): A 20-μm-deep chamber was prepared using polydimethylsiloxane (PDMS) and filled with photosensitive resin. The sensing probe was inserted into the chamber until it reached the bottom. With circle-patterned UV exposure and the progressive lifting of the probe, the resin was solidified layer by layer with a 10-μm layer thickness. The first five layers were fabricated in a circular pattern with a diameter of 200 μm to adhere tightly to the original sensing part. The remaining 95 layers were fabricated in a circular pattern with a diameter of 100 μm as the anchor for the artificial muscles. Thus, the sensing part of the probe was lengthened by approximately 1 mm, as the scanning electron microscopy (SEM) image shows. Based on the density of the resin (approximately 1.12 g/cm^3^), the mass of the lengthened part was approximately 0.01 mg, which was too small to activate the transduction property of the sensor. However, due to the extension, the distance between the acting position of the force and the location of the movable capacitor plates increased compared to the original sensor, introducing an added moment to the sensor and changing the force transduction coefficient. Therefore, it was necessary to calibrate the sensor before use.

### Preparation of artificial muscle solution

Three molecular motors made up the self-assembling artificial muscle: CaMLMM, K465m13, and microtubules. CaMLMM was a fusion protein of calmodulin (CaM) and light meromyosin (LMM), purified following a protocol similar to the purification of skeletal muscle myosin. K465m13 was a fusion protein of kinesin-1 and calmodulin-binding sequence m13, purified using a previously reported method for His-tagged protein expressed in *Escherichia coli* [30]*.* Microtubules were polymerized from fluorescent-labeled tubulin, which was purified from porcine brains following the HMPB (high molarity PIPES buffer) tubulin purification protocol [31].

The photoactivatable artificial muscle solution was prepared by mixing 4 μM K465m13 (0.48 mg/ml), CaMLMM (0.44 mg/ml), and microtubules (0.40 mg/ml) in a low salt buffer (20 mM tris-acetate, pH 8.0, 100 mM potassium acetate, 14 mM magnesium sulfate, 10 mM dithiothreitol, 0.01% Triton X-100, 10 mM adenosine triphosphate, 50 μM paclitaxel) containing caged-Ca^2+^ reagents [0.5 mM o-nitrophenyl EGTA, tetrapotassium salt (NP-EGTA N6802, Invitrogen), 0.1 mM CaCl_2_].

### Contractile force measurement

The procedure for measuring the contractile force generated by the artificial muscle is illustrated in Fig. 2D. Before measurement, the sensor was powered on for 1 h to achieve a stable operational state, and the position of the stage was adjusted according to the chamber height to focus the laser in the center of the microchamber. First, a sufficient amount of artificial muscle solution was pipetted into the microchamber to fill the first layer. Then, the mineral oil was carefully dropped into the second layer covering. The sensing probe was then inserted into the microchamber. There was a distance of 50 μm between the sensing probe and the bottom of the microchamber, and the distance between the fixed pillar and the sensing probe was controlled at 1 mm. The zero-point drift of the sensor caused by the buoyant force and surface tension of the solution was then removed using the software system. Accordingly, the sensor could measure the force relative to this initial state, namely, the contractile force of the artificial muscles. Finally, the UV laser was controlled to scan a rectangular pattern (*l* × *w*) in the microchamber; and contractile artificial muscles were formed in the irradiated area and wrapped the pillar and sensing probe. The force acting on the lengthened part of the sensing probe was measured and recorded using the software system.

## Results

### Calibration of the sensing probe

**Fig. 3.**
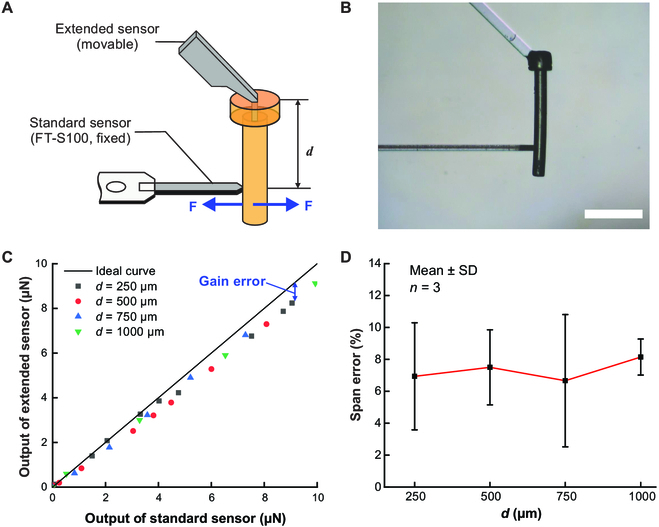
Calibration test and results. (A) Schematic diagram of the calibration test. The modified sensor was manipulated to contact the probe tip of a standard sensor, with its lengthened sensing part. The measured forces of the standard sensor were used to calibrate the modified sensor. (B) Image of the calibration test (*d* = 750 μm). (C) Outputs of standard and extended sensors with different *d* values. (D) Average gain error of the extended sensor as a function of *d*. Scale bar, 500 μm.

To evaluate the measurement accuracy of the modified sensing probe or the transduction coefficient of the force acting on the lengthened sensing part, we conducted calibration tests using another standard microforce sensing probe (FT-S100, FemtoTools), which had a higher measurement accuracy (resolution = 0.005 μN) along the probe axis. Since this sensor is difficult to anchor the artificial muscles that contract in its sensing direction, it is not suitable for measuring the contractile force of the artificial muscle. The standard sensor was horizontally fixed, and the extended sensor was manipulated to contact it with the lengthened sensing part (Fig. 3A and B). Theoretically, equal forces act on both sensors. The forces increased with the increasing proximity of the extended sensor and were synchronously measured by both sensors. We assumed the measured values of the standard sensor to be true values for calibrating the extended sensor and then tested it at different positions of the lengthened part. The distance between the contact point on the lengthened part and its top (denoted as *d*) varied from 250 to 1,000 μm.

Figure 3C shows the results of the calibration test. The outputs displayed a linear relationship, but the slopes of the calibration curves were smaller than that of the ideal curve; there were negative gain errors in the force transduction of the extended sensor. We repeated the calibration test and calculated the average gain errors for different *d* values, as presented in Fig. 3D. The gain error had no marked relationship with *d*, implying that within reasonable limits, the force transduction property of the sensor was not affected by the moment applied to the sensing probe. We attributed this robustness to the U-shaped microplanar springs connecting the movable capacitor plates that had a relatively strong bending resistance to the bending moment acting on them. The average gain error of 7.15% was used to correct the extended sensor.

### Measurement of contractile force

Contractile force measurements were first performed in a microchamber with a first layer height of 710 μm. To evaluate the relationship between the contractile force of the artificial muscles and the dimensions of the UV illumination area, the muscles were activated by scanning a rectangular pattern with a length of 3 mm and widths (denoted as *w*) of 500, 1,000, and 1,500 μm. A control experiment was conducted under the same conditions without UV scanning.

**Fig. 4.**
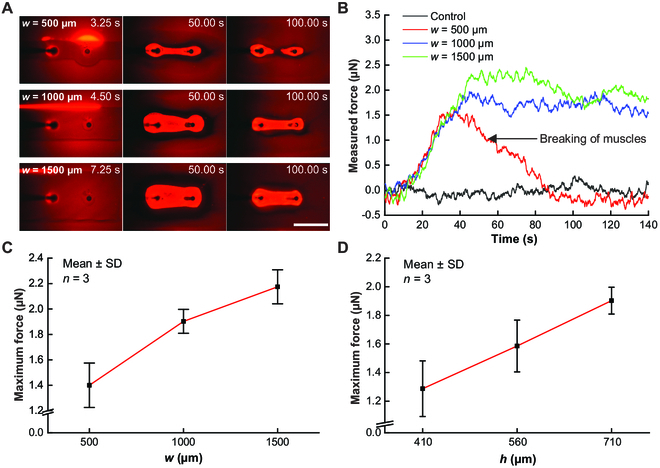
Contractile measurement results. (A) Contraction processes of artificial muscles (*h* = 710 μm and *w* = 500, 1,000, and 1,500 μm). (B) Profiles of measured forces with different *w* values (*h* = 710 μm). (C) Maximum force as a function of the width of the illumination area (*w*) when *h* was 710 μm. (D) Average maximum force as a function of the height of the first layer of the microchamber (*h*) when *w* was 1,000 μm. Scale bar, 1 mm.

The sequential fluorescent images in Fig. 4A (Movies S1 to S3) show the contraction processes of the artificial muscles. Figure 4B shows the measured forces within 140 s after initiating the UV scanning, with correction based on the calibration tests. The results showed that the force acting on the sensing probe gradually increased with the contraction of the artificial muscles and reached the maximum value when the muscles had almost contracted to the minimum shape. For *w* = 1,000 and 1,500 μm, the forces remained at the maximum level for an extended time because the artificial muscles continued to tightly link the sensing probe and the fixed pillar; for *w* = 500 μm, the force soon fell from the maximum level to zero because the artificial muscles ruptured. When the artificial muscles were activated with a wider illumination area, more time was required for contraction and a larger maximum force was achieved. The relationship between the maximum force and width of the illumination area is shown in Fig. 4C. When *w* = 500, 1,000, and 1,500 μm, the average maximum forces were 1.40 ± 0.17, 1.90 ± 0.09, and 2.17 ± 0.13 μN, respectively.

With a wider illumination area, more molecular motors were activated, and a stronger artificial muscle chain was formed linking the pillars, which generated a larger tensile force to the anchored pillars; this chain was less likely to rupture. However, as the width continued to increase, a diminishing return on the force generated was observed. Measurements were carried out in the microchambers with the 410- and 560-μm-high first layers, wherein the artificial muscles were activated with a 1 × 3 mm UV illumination area. The relationship between the maximum force and *h* is shown in Fig. 4D. When *h* was 410, 560, and 710 μm, the average maximum forces were 1.29 ± 0.19, 1.59 ± 0.18, and 1.90 ± 0.09 μN, respectively. The maximum force of the artificial muscles increased almost linearly with the height of the solution.

**Fig. 5.**
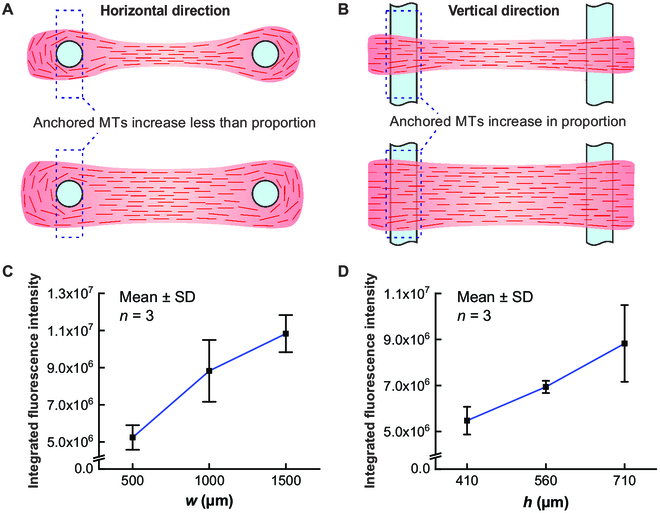
Comparison of the variation trend of the tensile force with the size of illumination space in different directions. (A) Less-than-proportional increase of anchored microtubules (MTs) (in the dotted box) with illumination width. (B) Proportional increase of anchored microtubules (in the dotted box) with illumination height. Average integrated fluorescence intensity as a function of the (C) width of the illumination area (*w*) and (D) height of the first layer of the microchamber (*h*).

We speculated that the different relationships between the tension force and size of the activated space in the horizontal and vertical directions were due to the different anchorage capacities of the pillars. Theoretically, the tension force and strength of the muscle chain largely depend on the weakest part of the chain, namely, the part with the least molecular motors (mainly microtubules). In many cases, the muscle chain ruptured on the sides of the pillars. Accordingly, we theorized that this area usually had the least number of microtubules and, therefore, dictated the strength of the entire chain. In the horizontal direction, the ability of the pillar to anchor microtubules was limited. When the width of the illumination area increases, the microtubules are more attracted to the center of the chain; thus, the quantity of anchored microtubules around the pillar (in the dotted box) increases less than proportionality (Fig. 5A). In the vertical direction, the capacity of the pillar to anchor microtubules could be expanded by increasing the thickness of the artificial muscle chain. Thus, when the height of the activated space increases, the anchored microtubules around the pillar (in the dotted box) increase proportionally (Fig. 5B).

To test this hypothesis, we calculated the integrated fluorescence intensity in a 100-μm-wide area on the sides of the sensing probe when *t* = 100 s, which reflected the quantity of microtubules within the area. The average integrated fluorescence curves as functions of *w* and *h*, which are similar to the maximum force curves, are shown in Fig. 5C and D, respectively. This image analysis provided preliminary evidence for our hypothesis. Because of the insufficient quantity of sample points in this work, which made our conclusion uncertain in consideration of possible errors, we will conduct more measurements to further verify our hypothesis.

### Tensile test of the artificial muscle

**Fig. 6.**
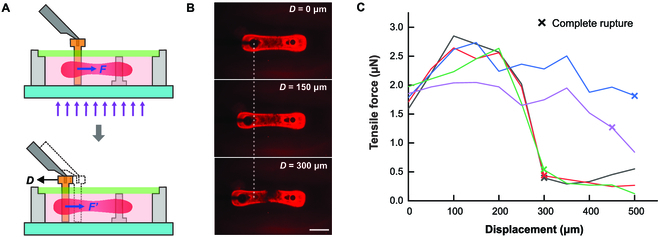
Tensile test and results. (A) Tensile test of the contracted muscle chain. (B) Images of the muscle chain with different displacements during the tensile test. (C) Tensile force–displacement curves in different experiments. Scale bar, 500 μm.

The mechanical properties of the contracted artificial muscle under tensile load were also evaluated using the proposed measurement system. As shown in Fig. 6A, the artificial muscle was first patterned in a 710-μm-high microchamber by a 1 × 3 mm UV illumination space. After it contracted to the minimum shape, the sensing probe was moved 500 μm to the left in steps of 50 μm, and the tensile force at each step was recorded. Figure 6B shows the process of the tensile test (Movie S4), in which the muscle chain was stretched as the sensing probe began to move. The muscle chain showed signs of rupture when the probe moved 150 μm and completely ruptured when the probe moved 300 μm.

The tension–displacement curves in Fig. 6C show the test results, the trend of which is similar to the stress–strain curves of ductile materials. The tensile force increased with elongation at first. It then began to decline after reaching a maximum similar to ultimate strength. We assumed that an elastic deformation took place during the first 100-μm displacement, and the cross-section of the muscle chain was a 214-μm-diameter circle since the width of the center of the muscle chain is nearly the same when viewed at different angles (Fig. S2). Thus, the stiffness of the muscle chain was estimated to be about 0.19 kPa.

After the muscle chain ruptured completely, the tension decreased sharply but did not immediately return fully to zero. This may be caused by the viscous resistance that had not completely disappeared at the moment of measurement, as the force only returned to zero after the probe had stopped moving for some time. The displacement for complete rupture of the muscle chain varied from 300 to 500 μm in different experiments.

## Discussion

In this study, we developed a measurement system and method to evaluate the contractility of artificial muscles directly using a capacitive microforce sensor extended by a 3D-printed microcantilever. The measurement accuracy of this sensor was ensured after calibration and correction. Contractile measurements and tensile tests were successfully performed using the proposed method, and the performance of the artificial muscle as a bio-actuator was further demonstrated. Currently, the artificial muscle is still far less powerful than engineered skeletal muscles in terms of peak stress and contraction rate, and cannot be operated at a high frequency, while it has unique advantages in contractile strain and activation mode (Table S1). The results also indicated that the contractile force of the artificial muscles increased with the size of the space where the molecular motors were activated, and the increase in the space in the direction along the wrapped pillar improved the contractile force more than that in the direction perpendicular to the pillar. Although the tensile properties could not be accurately quantified due to the differences in tensile test results, the results indicated that stretching no more than 10% of the muscle chain length can improve its contractility, while stretching more than 30% would cause a rupture of the muscle chain. These discoveries will help us utilize the artificial muscle more efficiently.

Compared with the cantilever-bending method used in our previous works [15,17], the contractile measurement in this work avoids manufacturing cantilever beams, measuring the elastic modulus of cantilever beams, and image processing. The force–time curve measured by the cantilever-bending method rose slower, which was due to the slower bending of the cantilever to achieve force equilibrium. In addition, there was significant variability in the measured results after the manufacturing of the cantilever beam due to degradation, which our current methodology avoids. Moreover, the proposed method allows for measuring additional mechanical properties like the tensile performance of the artificial muscles through simple operations. These indicate the efficiency, real-time responsiveness, stability, and flexibility of the proposed method. This work provides a useful tool for analyzing the contractility of our artificial muscles, as well as other types of emerging molecular motor-based artificial muscles [13,14]. Through this method, we can further explore the various mechanical properties of the artificial muscle and the associated influencing factors to support its application in biohybrid robotics.

## Data Availability

All data underlying the results are available within the article and its supplementary materials. Raw data that support the findings of this study are available from the corresponding author, upon reasonable request.
