# Supplementary material for: Contractile and Tensile Measurement of Molecular Artificial Muscles for Biohybrid Robotics
Source: Cyborg Bionic Syst. 2024 May 8;5:0106. doi: 10.34133/cbsystems.0106 (PMC11077706; doi:10.34133/cbsystems.0106)
Supplement: Supplementary 1 — Figs. S1 and S2 Table S1 Movies S1 to S4 [file cbsystems.0106.f1.zip › Supplementary_Materials.pdf]

Supplementary Materials for

Contractile and tensile measurement of molecular artificial muscles for biohybrid robotics

Yingzhe Wang, Kaoru Uesugi, Takahiro Nitta, Yuichi Hiratsuka, and Keisuke Morishima*

*Corresponding author. E-mail: morishima@mech.eng.osaka-u.ac.jp

This file includes:

Fig. S1. Conceptual diagram of biomolecular artificial muscle.
Fig. S2. Images of the contracted artificial muscle taken from different angles during a tensile test (h = 710 μm and w = 1000 μm).
Table S1. Comparison of biomolecular artificial muscle and engineered skeletal muscle.

Other Supplementary Material for this manuscript includes the following:

Movies S1 to S3: Contractile measurement processes of the artificial muscles (h = 710 μm and w = 500 μm, 1000 μm and 1500 μm).
Movies S4: Tensile test process of the artificial muscles (h = 710 μm and w = 1000 μm).

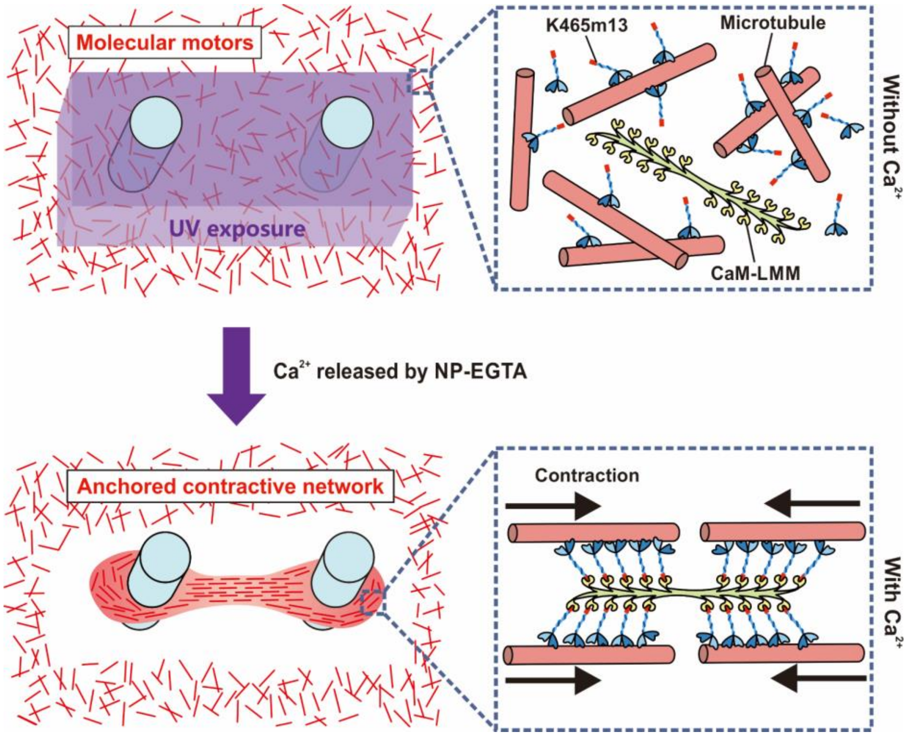

Fig. S1. **Conceptual diagram of biomolecular artificial muscle.** The artificial muscle consists of microtubules and two genetically engineered fusion proteins, CaMLMM, a fusion protein of calmodulin (CaM) and light meromyosin (LMM), and K465m13, a fusion protein of kinesin-1 and calmodulin-binding sequence m13. UV exposure in a designated area increases local calcium ion concentration. Calcium ions induce the binding of K465m13 to CaMLMM filaments, forming kinesin filaments. Kinesin filaments induce the sliding of microtubules, leading to dynamic self-assembly of a contractile network and macroscopic contraction of the artificial muscle.

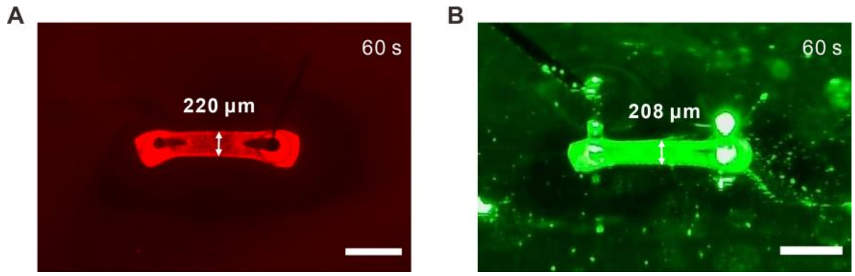

Fig. S2. **Images of the contracted artificial muscle taken from different angles during a tensile test (*h* = 710 μm and *w* = 1000 μm). (A)** Top-view image. **(B)** 45° oblique image. Scale bars: 500 μm.

Table S1. Comparison of biomolecular artificial muscle and engineered skeletal muscle.

	**Peak stress (kPa)**	**Stiffness (kPa)**	**Maximum strain (%)**	**Strain rate (%·s^{-1})**	**Activation mode**	**Energy source**
**Artificial muscle (this work)**	<0.1	~0.19	>80	~0.02	Photo-printing	ATP
**Engineered skeletal muscle**	0.56–10 [1]	10–40 [2]	5–20 [3, 4]	~5	Electrical/light stimulation	Glucose

References

1. Ricotti, L., B. Trimmer, A. W. Feinberg, R. Raman, K. K. Parker, R. Bashir, M. Sitti, S. Martel, P. Dario and A. Menciassi. "Biohybrid actuators for robotics: A review of devices actuated by living cells." *Science Robotics* 2 (2017): eaaq0495.
2. Collinsworth, A. M., S. Zhang, W. E. Kraus and G. A. Truskey. "Apparent elastic modulus and hysteresis of skeletal muscle cells throughout differentiation." *American Journal of Physiology-Cell Physiology* 283 (2002): C1219-C27.
3. Morimoto, Y., H. Onoe and S. Takeuchi. "Biohybrid robot powered by an antagonistic pair of skeletal muscle tissues." *Science Robotics* 3 (2018): eaat4440.
4. Raman, R., C. Cvetkovic, S. G. Uzel, R. J. Platt, P. Sengupta, R. D. Kamm and R. Bashir. "Optogenetic skeletal muscle-powered adaptive biological machines." *Proceedings of the National Academy of Sciences* 113 (2016): 3497-502.
